# Influence of early-life body mass index and systolic blood pressure on left ventricle in adulthood – the Cardiovascular Risk in Young Finns Study

**DOI:** 10.1080/07853890.2020.1849785

**Published:** 2020-12-14

**Authors:** Jarkko S. Heiskanen, Jussi A. Hernesniemi, Saku Ruohonen, Nina Hutri-Kähönen, Mika Kähönen, Eero Jokinen, Päivi Tossavainen, Merja Kallio, Tomi Laitinen, Terho Lehtimäki, Jorma Viikari, Markus Juonala, Jaakko Nevalainen, Olli T. Raitakari

**Affiliations:** a Research Centre of Applied and Preventive Cardiovascular Medicine, University of Turku, Turku, Finland; b Centre for Population Health Research, University of Turku and Turku University Hospital, Turku, Finland; c Tays Heart Hospital, Tampere University Hospital and Faculty of Medicine and Health Technology, Tampere University, Tampere, Finland; d Orion Pharma, Espoo, Finland; e Department of Paediatrics Tampere University Hospital and Faculty of Medicine and Health Technology, Tampere University, Tampere, Finland; f Department of Clinical Physiology, Tampere University Hospital and Faculty of Medicine and Health Technology, Tampere University, Tampere, Finland; g Department of Paediatric Cardiology, HUS New Children’s Hospital, University of Helsinki, Helsinki, Finland; h Department of Pediatrics, PEDEGO Research Unit and Medical Research Center Oulu, Oulu University Hospital and University of Oulu, Oulu, Finland; i Department of Clinical Physiology, University of Eastern Finland and Kuopio University Hospital, Finland; j Department of Clinical Chemistry, Fimlab Laboratories, and Finnish Cardiovascular Research Center - Tampere, Faculty of Medicine and Health Technology, Tampere University, Tampere, Finland; k Department of Medicine, University of Turku and Division of Medicine, Turku University Hospital, Turku, Finland; l Health Sciences Unit, Faculty of Social Sciences, Tampere University, Tampere, Finland; m Department of Clinical Physiology and Nuclear Medicine, Turku University Hospital, Turku, Finland

**Keywords:** Left ventricular mass, body mass index, blood pressure, risk factor, epidemiology

## Abstract

**Background:**

Increased left ventricular mass (LVM) predicts cardiovascular events and mortality. The objective of this study was to determine whether early-life exposures to body mass index (BMI) and systolic blood pressure (SPB) affects the left ventricular structure in adulthood.

**Methods:**

We used longitudinal data from a 31-year follow-up to examine the associations between early-life (between ages 6–18) BMI and SPB on LVM in an adult population (*N* = 1864, aged 34–49). The burden of early-life BMI and SBP was defined as area under the curve.

**Results:**

After accounting for contemporary adult determinants of LVM, early-life BMI burden associated significantly with LVM (3.61 g/SD increase in early-life BMI; [1.94 − 5.28], *p* < 0.001). Overweight in early-life (age- and sex-specific BMI values corresponding to adult BMI > 25 kg/m^2^) associated with 4.7% (2.5–6.9%, *p* < 0.0001) higher LVM regardless of BMI status in adulthood. Overweight in early-life combined with obesity in adulthood (BMI > 30kg/m^2^) resulted in a 21% (17.3–32.9%, *p* < 0.0001) increase in LVM. Higher early-life BMI was associated with a risk of developing eccentric hypertrophy. The burden of early-life SPB was not associated with adult LVM or left ventricular remodeling.

**Conclusions:**

High BMI in early-life confers a sustained effect on LVM and the risk for eccentric hypertrophy independently of adulthood risk factors.

**KEY MESSAGES**
1. Excess in BMI in early-life has an independent effect on LVM and the risk of developing eccentric hypertrophy regardless of overweight status in adulthood.
2. Systolic blood pressure levels in early-life did not have an independent effect on LVM or LV remodeling.
3. The clinical implication of this study is that primary prevention of obesity in early-life may prevent the development of high LVM and eccentric hypertrophy.

## Introduction

Obesity and hypertension are the major modifiable risk factors for increased left ventricular (LV) remodeling and LV mass (LVM) in adults [1]. In addition, age, male sex, diabetes, metabolic syndrome, alcohol consumption, and intense athletic training are associated with higher LVM [2–4]. Increased LVM is manifested by different LV remodeling patterns defined as eccentric hypertrophy, concentric hypertrophy, and concentric remodeling [5]. Increased LVM and LV remodeling are associated with an increased risk of heart failure and mortality in adults [6–8].

It is well established that excess body weight in childhood is associated with higher LVM in children, and it predicts increased LVM in young adulthood [9,10]. Furthermore, children with elevated blood pressure have higher LVM compared to normotensive children [11]. These often co-existing factors, excessive childhood adiposity, and elevated blood pressure are also associated with LV remodeling already in childhood in obese and hypertensive populations [10,12]. Previously, in a study by Lai et al., childhood body mass index (BMI) and systolic blood pressure (SBP) were associated with higher LVM and with the risk of eccentric and concentric hypertrophy in adulthood [13]. However, this study was done in an overweight population with participants on antihypertensive therapy excluded. In addition, other known risk factors, e.g. alcohol usage and physical activity, were not assessed. It is unclear if increased BMI or elevated SBP in childhood and adolescence can have long-lasting effects on LVM or LV remodeling in a general Caucasian population independent of adulthood risk-factors.

Childhood obesity is a growing global problem; therefore, it is of interest to examine its long-term health effects. Possible adverse effects on adult health would warrant more emphasis on primary prevention already from childhood. Thus, this study aimed to determine whether early-life exposures to BMI and SBP affect the LVM and LV remodeling in adulthood, independently of adulthood risk factors of LVM.

## Materials and methods

**Figure 1.**
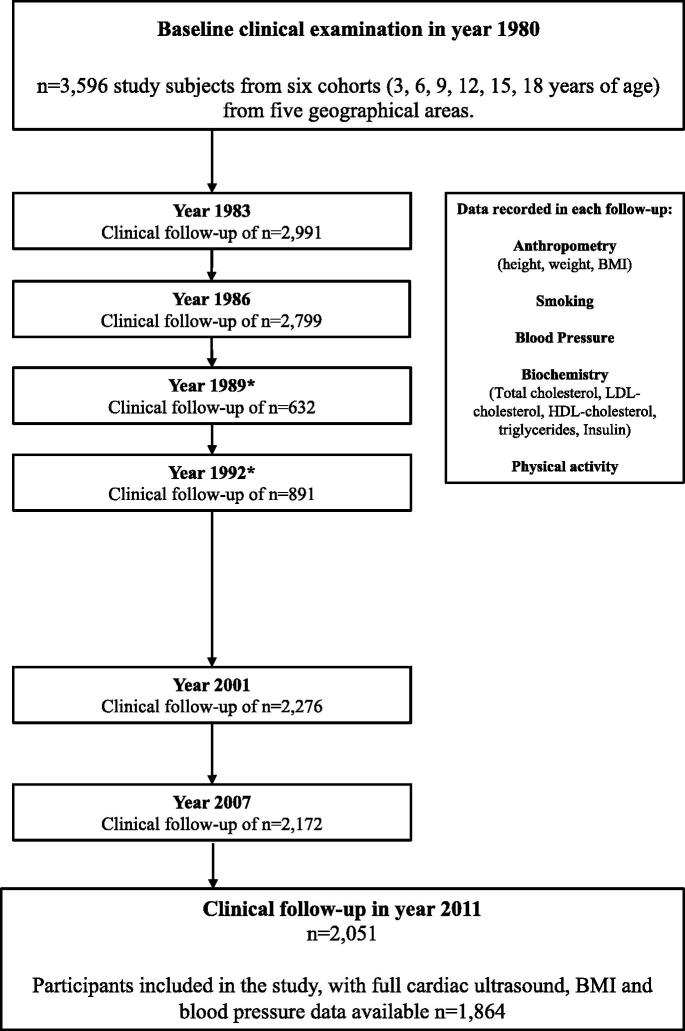
Flowchart of the on-going Cardiovascular Risk in Young Finns Study *Participants invited from only one of the five geographical areas.

We used the data of cardiac ultrasound measurements of the on-going multicenter study – the Cardiovascular Risk in Young Finns Study [14]. The Cardiovascular Risk in Young Finns Study was initiated in 1980 when participants were randomly recruited to six age cohorts of (3, 6, 9, 12, 15, and 18 years) from the national registry. Data on cardiovascular risk factors (anthropometric data, including BMI, physiological measurements such as blood pressure, biochemical measurements) were recorded. Physical activity and alcohol consumption (standard drinks/day) were collected using validated questionnaires. For additional information on the physical activity questionnaire, please see the online supplement. Follow-ups were performed in 3-year intervals until 1992 (1980, 1983, 986, 1989, and 1992), and additional follow-ups with more extensive measurements were organised in 2001 and 2007 (Figure 1) [15]. In the year 2011, echocardiography was performed for 1994 participants (914 men and 1080 women, mean age 41.9 ± 5.0 years). After excluding 11 women due to pregnancy at the time of cardiac ultrasound, participants with insufficient longitudinal data for BMI, SBP, and cardiac ultrasound measurements, the study population of the present study consisted of *n* = 1864 participants. Participants who were lost to follow-up between 1980 and 2011 were more often men (51% vs. 43%, *p* < 0.001), but there were no statistically significant (*p* > 0.05) differences between participants and non-participants in baseline age, BMI, or blood pressure values among men or women. By 2011, 93 of the original participants had died, with only 14.0% (*n* = 13) of the deaths attributable to cardiovascular causes. The study protocol conforms to the ethical guidelines of the 1975 Declaration of Helsinki and is approved by the ethics committee of the University of Turku. Informed consent was obtained from all participants. Specific details of the methodology have been described earlier [14,15].

The examinations were conducted according to American and European guidelines [5]. Trained ultrasound technicians performed the echocardiographic examinations at five study centres in Finland. All ultrasound technicians were trained by a cardiac imaging specialist. Transthoracic echocardiography was performed with Acuson Sequoia 512 (Acuson, Mountain View, CA) ultrasonography, using a 3.5 MHz scanning frequency phased-array transducer. Analysis of the echo images was done by one observer blinded to the clinical details with CommPACS 10.7.8 (MediMatic Solutions, Italy) analysis program. The methodology for imaging has been described earlier in more detail [16]. LVM and relative wall thickness were calculated from M-mode parasternal long-axis views based on diastolic values. LVM was indexed to body height (m^2.7^) as the LVM index expressed in g/m^2.7^ [17]. LV end-diastolic volume was measured from apical four-chamber view images with Simpson’s rule in a single plane.

To evaluate the possible association of the long-term burden of BMI and systolic blood pressure in early-life (between ages 6 and 18) on LVM in adulthood, area under the curve values (AUC) were calculated for each participant by using the results from repeated measurements of BMI and SBP and used as main exposure variables. Participant-specific curves for BMI and blood pressure were estimated by mixed model regression splines [18]. For more detailed information on the methodology, please see Online supplement 1. Similar to the approach of Lai et al. [13], we then evaluated the AUC as a measure of a long-term burden of each of the measured attributes. The most extensive set of measurements was available between ages 6 and 18 years, after which the proportion of measurements decline significantly. The AUC values were standardised for analysis (z-transformed with mean = 0 and standard deviation (SD)=1). All the effect sizes are reported as an absolute change in the response variable (in grams) corresponding to one SD increase in exposure variables with continuous distribution unless stated otherwise.

The main outcome variable was LVM, and secondary endpoints were LV volume and LV remodeling patterns: eccentric hypertrophy (LV hypertrophy with low relative wall thickness), concentric hypertrophy (LV hypertrophy with high relative wall thickness), and concentric remodeling (high relative thickness without LV hypertrophy). The remodeling phenotypes were defined by using the population 85th cut-off values for LVM and relative wall thickness. We used multivariable linear adjusted regression analysis to analyse the association between main exposure variables and outcome variables. All the analyses were adjusted for age, sex, adult BMI, and adult SBP. We also included the study centre as a technical covariate in the statistical models to ensure that the results are not driven by differences between the study centres. Alcohol consumption and physical activity in adulthood were significantly associated with LVM (*p* <0.05 for both) and were used as covariates in the multivariable models. To verify the magnitude of observed associations and correct for possible issues due to collinearity and multicollinearity in regression analysis, we calculated inverse probability weights to fit marginally structural models. These models are used to estimate causal effects from observational data by correcting for confounding with minimal risk for adjusting away part of the effect [19]. R-package ipw was applied in the analysis (http://CRAN.R-project.org/package=ipw).

**Figure 2.**
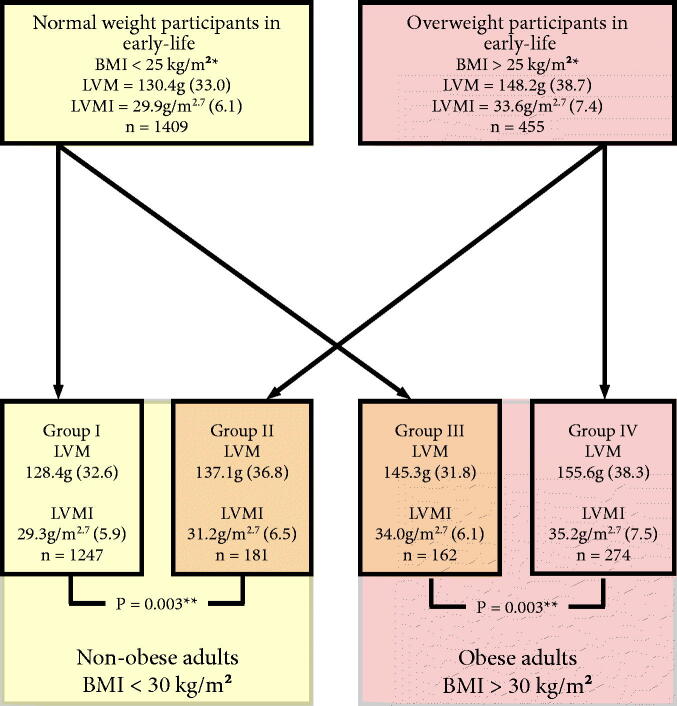
Adulthood LVM by BMI development from early-life to adulthood. Numbers in parentheses correspond to standard deviation values unless stated otherwise. *Participants were defined as overweight in early-life if: (I) their age- and sex-specific BMI values exceeded the international BMI percentiles corresponding to adult BMI of 25 kg/m^2^ in at least 50% of the measured values between ages 6 and 18 or (II) their BMI burden between years 6 and 18 calculated as AUC exceeded AUC value derived from the international BMI corresponding to adult BMI of 25 kg/m^2^. Group I: non-overweight in early-life and non-obese in adulthood, Group II: overweight in early-life and non-obese in adulthood, Group III: non-overweight in early-life and obese in adulthood, Group IV: overweight in early-life and obese in adulthood. **Unadjusted *p*-value for comparison of LVM between the groups. Abbreviations: AUC: area under curve; BMI: body mass index; LVM: left ventricular mass; LVMI: left ventricular mass indexed for height^2.7^.

For stratified analysis, the study population was stratified into four groups by overweight status in early-life and obesity in adulthood at follow-up in 2011 (Figure 2). Participants were defined as having excess early-life overweight if (I) their age- and sex-specific BMI values exceeded the international cut off points for BMI (defined by Cole et al.) corresponding to adult BMI of 25 kg/m^2^ in at least 50% of the measured values between ages 6 and 18 or (II) their BMI burden between years 6 and 18 calculated as AUC exceeded AUC value derived from the international cut off points for BMI corresponding to adult BMI of 25 kg/m^2^ [20]. Adult participants were stratified by obesity status using a cut-off of 30 kg/m^2^.

Confidence intervals (CI) are reported as 95% CI. We used all available data in the analyses. Therefore, the number of participants varies between the models. Data were analysed with the R statistical package, version 4.0.2 (R Core Team (2016). R: A language and environment for statistical computing, R Foundation for Statistical Computing, Vienna, Austria, http://www.R-project.org/).

Patients or the public were not involved in the design, conduct, reporting, or dissemination plans of our research.

## Results

Study population characteristics at the end of the follow-up (in 2011) are presented in Table 1. The study population represents the general Caucasian population. At the baseline of the study, the prevalence of the participants exceeding the international BMI percentiles corresponding to adult BMI of 25 kg/m^2^ was 7.3%. The overall prevalence of obesity (BMI ≥ 30 kg/m^2^) in adulthood was 19.4%. Mean LVM was significantly higher in men (158.2 g) than in women (115.2 g). We did not find evidence for sex-specific differences in the associations between early-life body mass index and systolic blood pressure with adult LVMI (data not shown). LVM in adulthood was significantly associated with BMI, SBP, and age (Table 2). Diabetes, daily smoking, diastolic blood pressure, use of blood pressure-lowering medication, and metabolic syndrome did not associate significantly with LVM (*p* > 0.05 for all) when the analysis was adjusted for BMI and sex.

**Table 1.** Characteristics of the study participants in adulthood (measured in 2011) with full cardiac ultrasound data.

	Men (*n* = 846)	Women (*n* = 1018)
Age, years (range 34–49 years)	41.7 (5.0)	42.1 (4.9)
Diabetic (%) (*n* = 10 with Type I)	31 (3.7)	35 (3.4)
Daily Smokers (%)	134 (15.8)	130 (12.8)^‡^
Body-Mass Index, kg/m^2^	26.7 (4.0)	26.0 (5.4)^‡^
Overweight* (%)	537 (63.4)	506 (49.7)^‡^
Obese^† ^(%)	165 (19.5)	197 (19.4)
Systolic blood pressure, mmHg	122.9 (13.4)	115.5 (13.6)^‡^
Diastolic blood pressure, mmHg	77.5 (10.8)	72.3 (9.5)^‡^
Physical activity (index score ranging 5–15)^§^	9.0 (1.9)	9.15 (1.9)
Alcohol consumption (drinks/day)^§^	1.2 (1.5)	0.51 (0.73)^‡^
Use of blood pressure lowering medication (*n*)	72 (8.5)	87 (8.5)^‡^
Metabolic disorder (%)	199 (23.5%)	177 (17.4%)^‡^
Left Ventricle Mass, g	158.2 (32.8)	115.2 (23.5)^‡^
Left Ventricle Mass Index, g/m^2.7^	32.5 (6.8)	29.4 (6.1)^‡^
Relative Wall Thickness	0.286 (0.038)	0.278 (0.037)^‡^

*Overweight: adult body mass index ≥ 25kg/m^2 †^Obese: adult body mass index ≥ 30 kg/m^2 ‡^*p* < 0.05 for the comparison between men and women. ^§^men (*n* = 747), women (*n* = 935).

Values are presented as mean ± SD for the continuous variable and n (%) for categorical variables.

**Table 2.** Regression analyses of factors associating with the left ventricular mass in the study population.

	Adulthood model 1^†^			Combined model^†^		
	Coefficients	CI	*p*-value	Coefficients	CI	*p*-value
Age (years)	0.35	0.11 − 0.60	0.005	0.42	0.17 − 0.68	0.001
Male sex	39.93	37.35 − 42.51	<0.001	39.99	37.41 − 42.57	<0.001
Adult BMI, kg/m^2^	12.04	10.69 − 13.39	<0.001	10.01	8.39 − 11.62	<0.001
Adult SBP, mmHg	2.16	0.78– 3.54	0.002	2.08	0.59–3.57	0.006
Early-life BMI*	–		–	3.61	1.94 − 5.28	<0.001
Early-life SBP*	–		–	0.42	−1.04 − 1.89	0.570
Variance Explained:	50.7 %			51.5 %		

*Both Early-life BMI and Early-life SBP were measured as the area under the curve derived from longitudinal measurements between the ages 6 and 18.

^†^Models were additionally adjusted for study centre in the year 2011 adult alcohol consumption and physical activity.

The coefficients indicate the change in the outcome variable (measured in grams) corresponding to one standard deviation change in continuous exposure variables or for the difference between sexes.

Abbreviations: BMI: body mass index; CI: Confidence interval; SBP: systolic blood pressure.

In multivariable models, after accounting for adult risk factors, early-life BMI associated significantly with LVM (3.61 g per one SD in BMI; CI 1.94 − 5.28, *p* < 0.001). This association’s magnitude is ∼36% of the association between adult BMI and LVM (Table 2). No statistically significant changes were observed in the multivariate model when adjusting either with cumulative childhood physical activity, a blood pressure-lowering medication, having diabetes in adulthood, or birth weight (data not shown).

The Marginal Structural Model analysis gave similar estimates for both early-life BMI (5.23 g per one SD in BMI; CI 2.07–8.40g, *p* =0 .001) and adult BMI (11.65 g per one SD in BMI; CI 9.41–13.89g, *p* < 0.001). To analyse weight and height separately, we repeated the analysis by replacing early-life BMI with early-life weight and height (similar AUC estimates) and adjusted the analyses with adult height and weight. The independent association of early-life weight with LVM was substantial (4.18 g per one SD in weight; CI 2.02–6.34g, *p* < 0.001), whereas the early-life height was not associated with adulthood LVM (*p* = 0.14).

In the secondary analysis, early-life BMI was associated independently with the risk of developing an eccentric hypertrophy remodeling pattern. The increased risk for eccentric hypertrophy attributable independently to early-life BMI can be demonstrated by dividing the population into equal size quartiles by sex-specific early-life BMI values using the 1st quartile as the reference group. The odds ratio increased systematically between the early-life BMI quartiles, showing a significant difference between 1st and 3rd (*p* = 0.006), 1st and 4th (*p* = 0.001), and for trend (*p* < 0.001) across the groups (Table 3.). A similar analysis was conducted for concentric remodeling and concentric hypertrophy odds ratio between early-life BMI quartiles and the risk for eccentric hypertrophy. However, early-life BMI was not associated independently with the risk of developing concentric remodeling (*p* = 0.26 for trend across groups) or concentric hypertrophy (*p* = 0.057 for trend across groups). The independent association of early-life BMI on LV end-diastolic volume was highly significant and comparable to the effect of adult BMI (8.01 ml per one SD in early-life BMI; CI 6.43–9.60 ml, *p* < 0.0001 and 9.92 ml per one SD in adult BMI; CI 8.49–11.35.ml, *p* < 0.0001). Early-life SBP did not associate with adulthood LVM or any remodeling patterns after adjusting for adult risk factors (*p* > 0.05 for all analyses).

**Table 3.** Odds ratio between early-life BMI quartiles and the risk for eccentric hypertrophy in adulthood.

	2nd Quartile	3rd Quartile	4th Quartile
Odds ratio	1.00	1.8	2.24
Confidence interval	0.63–1.59	1.19–2.75	1.49–3.42
*p*-value	0.99	0.006	0.001

The population was divided into equal size quartiles by sex-specific early-life BMI. 1st quartile was used as the reference group. *p* < 0.001 for trend across groups.

To illustrate the independent association of early-life high BMI and the combined effects of high BMI in early-life and adulthood, we stratified the study population into four groups by overweight status in early-life and obesity in adulthood at follow-up in 2011 (Figure 2).

Before adjusting for other factors, participants who had been overweight in early-life had ∼14% higher LVM in adulthood among non-obese and obese adults (Figure 2). After accounting for adult BMI and other adult determinants, early overweight was associated with 4.7% (CI 2.5–6.9%, *p* < 0.0001) higher LVM in the whole study population.

The additive effect of high BMI from early-life to adulthood can be demonstrated by comparing participants who were continuously exposed to high BMI from early-life to adulthood (Group IV) to participants without the exposure to high BMI (Group I) The unadjusted difference of 27.2 g in mean LVM values between these two groups is substantial (an increase of 21.2%, CI 17.3–32.9%) comparing Group I and Group IV, *p* < 0.0001). When the analysis was adjusted with all other adult determinants of LVM (as described earlier), the difference was slightly attenuated but remained highly significant (17.5%, 14.9–20.1%, *p* < 0.0001). Inline, continuous exposure to high BMI from early-life to adulthood associated with a higher risk for eccentric hypertrophy (adjusted odds ratio 2.04, CI 1.35–3.07, *p* < 0.001).

## Discussion

We found that the long-term burden of increased BMI in early-life is associated with increased LVM and the risk of developing eccentric type hypertrophy in adulthood regardless of adult BMI in the Young Finns Study cohort. This association remained strong even after accounting for adult SBP and BMI and other known adult determinants of LVM and was mainly attributable to higher absolute body weight.

Previously, a review article by Ghosh et al. [21] showed results of early-life high BMI resulting in worse cardiac structure but concluded that there is a lack of studies investigating the cumulative effect of life-course exposure to risk factors for cardiac structure. Our recent study addresses this gap in knowledge and demonstrates a strong association between the cumulative burden of early-life exposure to BMI on adult LVM and LV remodeling. To clarify the relevance of early-life exposure, we accounted for separately adult risk factors and other confounding factors related to excess body weight. Normal weight participants in early-life, who were non-obese in adulthood, had the lowest LVM. In contrast, participants with consistently high adiposity status (overweight or obese in early-life and obese in adulthood) had over 21% higher LVM. After accounting for adult BMI and other contemporary factors associated with LVM, being overweight in early-life resulted in 4.7% higher LVM in adulthood in the total study population. These observations are clinically relevant as increased LVM is an independent predictor of heart failure even in the absence of ischemic heart disease [22].

YFS has previously demonstrated that consistently high BMI from childhood to adulthood associated with a higher risk of type 2 diabetes, hypertension, adverse lipid status, and increased carotid intima-media thickness [23]. However, excess childhood adiposity did not seem to confer substantial risk for most of the outcomes among participants who became non-obese in adulthood [23]. Our recent study suggests that LVM is sensitive to early-life high BMI, and the effects last to adulthood. In addition, the results suggest that high BMI in early-life leads to a higher risk of eccentric hypertrophy regardless of BMI status in adulthood. Eccentric hypertrophy of the LV has been associated with obesity in adulthood, and a study by Lai et al. suggested that life course excessive adiposity affects the LV remodeling [13][24]. Although lifestyle changes in adulthood have also been shown to have a positive impact on LV geometry, our results support active intervention already in early-life overweight and obesity [26].

As an observational study, we lack a direct functional link between the burden of early-life BMI and LVM measured in adulthood. However, as high BMI increases total blood volume and cardiac output, the increased workload causes dilatation and stress leading to cardiac remodeling [25,26]. Supporting this, we observed that absolute body weight in early-life, which increases workload, has a strong and significant effect on LVM. The contributing pathophysiological mechanisms associated with increased workload may act through various pathways in the heart, including metabolic, endocrine, and inflammatory factors, ultimately leading to sarcomere replication and parallel growth of non-muscular myocardial components.[27] Our recent results suggest that the independent association of early-life BMI and LVM is shown mainly by the increased overall size of the ventricle by adapting its volume with a corresponding increase in LVM. Previous studies have associated obesity with concentric remodeling, but in their study populations, the study subjects have been morbidly obese with related comorbidities [28,29]. In our study, early-life BMI was associated with eccentric remodeling, in a study population comprising of a random sample of the general population with a low prevalence of extreme obesity (5.4% of participants with BMI ≥ 35 kg/m^2^ and 1.5% with BMI ≥40 kg/m^2^).

In this study, the increased burden of elevated SBP in early-life predicted higher LVM in adulthood, but the association was no longer significant when the analysis was adjusted for present risk factors, including adult SBP and BMI and early-life BMI. In the Beijing Blood Pressure Cohort Study, elevated blood pressure in childhood was seen to associate with the LVM index regardless of adult risk factor status [30]. However, childhood blood pressure and BMI were measured only on one occasion; thus, the variability of the parameters in childhood was not assessed. In addition, arbitrary criteria were used for childhood hypertension and adulthood obesity. In our study, we use cut-off values from international guidelines for categorised variables. Furthermore, cumulative childhood parameters are used, which captures the long-term effect of childhood risk factors more efficiently than a single measure. Thus, a direct comparison between these results cannot be made. Similar to our results, previous studies have shown that adulthood obesity may be a stronger risk factor for LV hypertrophy than elevated blood pressure or hypertension [24].

There are some limitations to our study that need to be discussed. One potential limitation is a possible selection of the study population. As in every longitudinal study, there is a loss to follow-up in the Young Finns Study. However, detailed assessments of the representativeness have previously demonstrated no significant differences between the participants and non-participants in the age and sex-adjusted analyses [14]. Comparisons between participants and those lost-to-follow-up using age-adjusted analysis have found no significant differences in either men or women in the major study variables, including anthropometrics, blood pressure, and serum lipoproteins. The Young Finns Study population is racially homogeneous; therefore, our results are generalisable to white Caucasian populations. Furthermore, since the echocardiography measurements have been measured thus far only once in our cohort, we could note evaluate longitudinal changes in LV hypertrophy in our population. Finally, to study the associations of cardiac remodeling and the burden of early-life BMI, we defined the remodeling phenotypes by using the population 85th cut-off values for LVM and relative wall thickness. Therefore, the number of participants in the various remodeling categories is higher than it would have been by using the clinical criteria of LV remodeling phenotypes based on the joint guideline by the American society of echocardiography and the European association of cardiovascular imaging [5]. The major strengths of this study include the longitudinal study design and the long follow-up of participants who were well phenotyped in childhood and adulthood.

In conclusion, we found that exposure to excess in BMI in early-life confers a sustained effect on LVM and the risk of developing eccentric hypertrophy in adulthood regardless of adulthood overweight status. The clinical implication of these findings is that primary prevention of obesity in early-life may prevent the development of high LVM and eccentric hypertrophy. As these traits are associated with a higher risk for heart failure even without the presence of ischemic heart disease, it is plausible that early-life high BMI could be an independent risk factor for heart failure.

## Data Availability

The data used is declared confidential. Enquires from the corresponding author.
